# Development and preliminary validation of an open access, open data and open outreach indicator

**DOI:** 10.3389/frma.2023.1218213

**Published:** 2023-09-07

**Authors:** Evgenios Vlachos, Regine Ejstrup, Thea Marie Drachen, Bertil Fabricius Dorch

**Affiliations:** ^1^University Library of Southern Denmark, University of Southern Denmark, Odense, Denmark; ^2^The Maersk Mc-Kinney Moller Institute, University of Southern Denmark, Odense, Denmark; ^3^Department of Physics, Chemistry and Pharmacy, University of Southern Denmark, Odense, Denmark

**Keywords:** open science, open access, open data, outreach, metrics, responsible use of indicators

## Abstract

We present the development and preliminary validation of a new person-centered indicator that we propose is named “OADO” after its target concepts: Open Access (OA), Open Data (OD) and Open Outreach (OO). The indicator is comprised of two factors: the research factor indicating the degree of OA articles and OD in research; and the communication factor indicating the degree of OO in communication activities in which a researcher has participated. We stipulate that the weighted version of this new indicator, the Weighted-OADO, can be used to assess the openness of researchers in relation to their peers from their own discipline, department, or even group/center. The OADO is developed and customized to the needs of Elsevier's Research Information Management System (RIMS) environment, Pure. This offers the advantage of more accurate interpretations and recommendations for action, as well as the possibility to be implemented (and further validated) by multiple institutions, allowing disciplinary comparisons of the open practices across multiple institutes. Therefore, the OADO provides recommendations for action, and enables institutes to make informed decisions based on the indicator's outcome. To test the validity of the OADO, we retrieved the Pure publication records from two departments for each of the five faculties of the University of Southern Denmark and calculated the OADO of 995 researchers in total. We checked for definition validity, actionability, transferability, possibility of unexpected discontinuities of the indicator, factor independence, normality of the indicator's distributions across the departments, and indicator reliability. Our findings reveal that the OADO is a reliable indicator for departments with normally distributed values with regards to their Weighted-OADO. Unfortunately, only two departments displayed normal distributions, one from the health sciences and one from engineering. For departments where the normality assumption is not satisfied, the OADO can still be useful as it can indicate the need for making a greater effort toward openness, and/or act as an incentive for detailed registration of research outputs and datasets.

## Introduction

During the last decade, researchers and institutions are increasingly being subjected to having their research outputs measured, compared, and evaluated. The Council of the European Union^1^ has recently extended an invitation toward promoting and rewarding Open Science (OS) considering it a mechanism for reinforcing research integrity and a driver for the quality and impact of science to the benefit of society. Similarly, the recent Coalition for Advancing Research Assessment (COARA) Agreement on Reforming Research Assessment Union^2^ calls institutions to develop and evaluate new assessment criteria and tools that will raise awareness, reward researchers, and inform policies on research outputs associated with openness. We align ourselves with the San Francisco Declaration on Research Assessment (2012) supporting the fact that for the purposes of research assessment and reward, the value and impact of more than one indicator and type of research output must be considered.

The concept of using OS for such purposes is not new as it has been used for country rankings (Zuiderwijk et al., 2021), for benchmarking at national levels (Harnad, 2009; Bracco, 2022; Chaignon and Daniel Egret, 2022), and at the introduction of incentive models to try to persuade researchers to implement OS (Till, 2003). The usefulness of Open Data (OD) has also been investigated, with the study of Abella et al. (2014) standing out as they introduced the Meloda metric for the indication of open data quality. Admittedly, the most common OS concept used for evaluations are counts or fractions or percentages of Open Access (OA) as Lnenicka et al. (2022) states. Interestingly, Maddi (2020) recognized that OA levels vary between subject fields and proposed the Normalized Open Access Indicator (NOAI). However, the NOAI is based off Web of Science subject categories, which poses an issue for a host of research fields not well indexed in Web of Science, or even in Scopus from which the metric could conceivably also be calculated.

In this study, we propose a new indicator that we suggest is called the “OADO” after its target concepts OA, OD and Open Outreach (OO). The indicator is comprised of two factors: a factor indicating the degree of OA and OD in research–and a factor indicating the degree of OO in communication activities. In the following sections, we will explain how the OADO functions, present the methodology for assessing the OADO function, and show our preliminary findings in relation to the OADO's validity, the possibility of unexpected discontinuities of the indicator, factor independence, normality of the indicator's distribution, and indicator reliability. Finally, we will discuss how and when it can be used, as well as address all detected limitations.

## Method

### The OADO function

The OADO function is a person-centered indicator that consists of two factors: the *research factor* indicating the degree of OA articles and OD in research, and the *communication factor* indicating the degree of OO in communication activities a researcher has. The incentive to break the function into these two factors is to raise researchers' awareness on the fact that apart from their efforts to make their research available, their effort to communicate it openly to the public also matters.

The OADO function is the following.


$$\begin{array}{l} OADO=a\frac{OA\_research\_publications\;+\;Open\_datasets}{ALL\_research\_publications}\\ \;+b\frac{OA\_communication\_publications}{ALL\_communication\_publications},\\ \;a,b\epsilon R\cap \left(0,1\right) \end{array}$$

The OADO is developed and customized to the needs of Elsevier's Research Information Management System (RIMS) environment, Pure.^3^ The definition of the “research publications” variable is following the Pure definition: “a product of a research activity that complies with the academic quality within the field and contributes to the development of the research field.” It includes any publication deemed as “research” by SDU, for example journal articles, conference proceedings, book chapters, monographs, scientific poster, and any other research articles. The “communication publications” variable is defined -according to Pure- as “a product that seeks to share research findings with a wider audience or stakeholders,” and it includes newspaper or magazine articles, websites or blog posts, documentary films and any other outreach/communication or policy articles. Open datasets are defined as data that follow the FAIR (Findable, Accessible, Interoperable, Reusable) principles (Wilkinson et al., 2016), and have as a minimum requirement a persistent identifier, a license, and a description. Research software and code are assimilated to data. The weights *a* and *b* are used to give more emphasis -if needed- either to the research, or the communication factor. For this study, we consider both factors of the equation to be of equal importance, thus *a* and *b* are equally weighted to one.

We follow a more pragmatic approach when considering the OADO assigning a dataset to a publication, just like SciVal monitors “Publications with datasets indexed in Data Monitor” under the Trends module. Perhaps this may change in the future if we take into consideration a quote from Barend Mons, one of the co-authors of the FAIR principles: “Now we have papers–meant for people-and behind the paper wall there is supplementary data. We have to turn that around and publish the research objects in their own right” (Deutz et al., 2020).

#### 2.1.1. The Weighted-OADO

The weighted version of this new indicator, the *Weighted-OADO*, takes into consideration the differences in OS practice of a researcher in relation to their peers from their own discipline, department, faculty, or even group/center. This offers the advantage of more accurate interpretations and recommendations for action, as well as the possibility to be implemented (and further validated) by multiple institutions, allowing disciplinary comparisons of the open practices across multiple institutes. The Weighted-OADO is a means to recognize the degree of OS and it should be calculated at the hierarchical level one wants to operationalize it.

If we consider the OADO at a department level, then we calculate the OADO for every researcher of that specific department, and then average all the OADO values of the researchers of the department to get the *Department Average OADO* value. Afterwards, we divide the OADO value of each researcher to the Department Average OADO to get the Weighted-OADO per researcher.


$$Weighted-OADO\;=\;\frac{OADO}{Department\_Average\_OADO}$$

A Weighted-OADO of a researcher that is: greater than one (>1), means that the researcher performs “better” than the Department Average OADO; equal to one (=1), means that the researcher performs exactly as the Department Average OADO indicates; less than one (< 1), means that the researcher performs below the Department Average OADO.

###  Implementing the OADO

**Figure 1:**
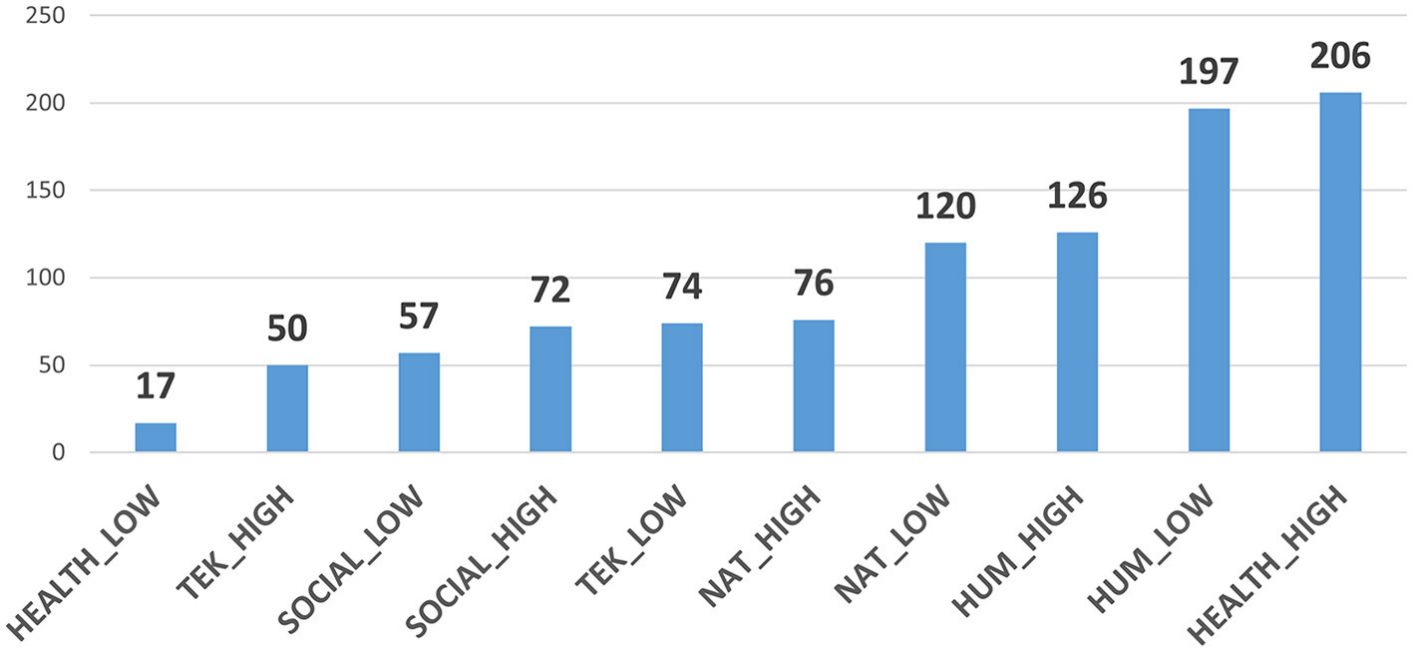
Number of researchers per department.

All data used for testing and validating the OADO are pulled from the University of Southern Denmark's (SDU) Pure system. We retrieved the Pure records from two departments for each of the five faculties of the SDU (Natural Science, Engineering, Health Science, Business and Social Science, Humanities). The departments were selected according to their performance (we selected the highest and the lowest performance per faculty) as indicated by the Danish Open Access Indicator^4^ which is released annually by the Danish Agency for Higher Education and Science, part of the Ministry of Higher Education and Science. It is collecting and analyzing publication data from all the Danish universities. Figure 1 shows the number of researchers per department varying from 17 to 206 persons, meaning that some departments perhaps focus only on one discipline while others may incorporate multiple disciplines.

#### Publication and dataset report in Pure

Publications are pulled from the Pure system taking into consideration the last 5 years (2018–2022) as 2018 was the year the SDU Open Science Policy^5^ was established. Publication categories are used to group publications into “research publications” or “communication publications” as mentioned earlier.

Publication category is set up as a required field when publications are entered in the RIMS database and is used to indicate intended readership of each publication. Researchers select a readership category themselves, however the final validation and possible corrections are made by a dedicated Pure team. Definitions of the categories are added on the template for registering publications to help researchers select correct categories. RIMS categories “education” and “commissioned” were not included in the data collection for the OADO.

For the OADO we include everyone currently affiliated to SDU who has at least one research or communication publication within the selected year span. At SDU, it is mandatory to add publications written during affiliation period. This is enforced by the university leadership and the library. It is voluntary, but encouraged, to add publications published during external or previous affiliation. Both publications from internal and external affiliations are included in the OADO when they are added in the publication database. In Pure, you are also allowed to register publications that are in preparation, however they are not taken into consideration when calculating the OADO.

Datasets are not uploaded directly in Pure, therefore only metadata entries that describe actual datasets can be found. All dataset entries from the last 5 years (not counting current year) are pulled from everyone currently affiliated to SDU.

#### Open access in Pure

To be considered OA, a publication needs to have either a valid link to an OA version of the article, a persistent identifier (for example, a digital object identifier), or have an available file attached to the publication entry in Pure. That file can be the final published version, the pre-print (a version of a manuscript prior to peer-review), or the post-print (the peer-reviewed version before editorial typesetting). Publications are counted as open despite being “under embargo” if there is an uploaded file that will become open in the future. Links to ambiguous, or unknown pages are being filtered out by a dedicated Pure Team. In general, the way the OADO identifies OA is similar to the service Unpaywall^6^ where any OA version is counted.

The process of registering research publication as OA in Pure is done primarily by a dedicated Pure Team. However, researchers can also add links and files themselves which are later checked by the Pure Team. In relation to OA communication publications, it is important to note that they are made available by the researchers themselves and are more challenging to verify, thus handled much more inconsistently than OA research publications.

### Validation criteria

A classic approach to validating an indicator is to compare it with other well known “established” measures, and more often than not this is an incorrect predictor (Roche, 1994). In our case, the OADO is a person-centered indicator attributed to the researcher, not the product of the research (e.g., an article). The -perhaps- most generally accepted indicator that is attributed to the researcher is the h-index (Hirsch, 2005), which is borderline irrelevant to what we wish to measure with the OADO. Therefore, we resorted to examining validation criteria stemming from the computer science domain.

First, we will graphically present the data using histograms and then we will present their quantile-quantile (Q-Q) plots (Wilk and Gnanadesikan, 1968) to get an estimation of which departments are normally distributed. Q-Q plots sort the data in ascending order, and then plot them vs. quantiles calculated from a theoretical distribution. If the data is *normally distributed*, the points in a Q-Q plot will lie on a straight diagonal line. To further validate our assumptions from the Q-Q plots we compute the Shapiro-Wilk test for normality (Shapiro and Wilk, 1965).

According to Roche (1994), one of the analytical principles that a measurement should adhere to is *factor independence* in case the indicator is composed of several factors. Factor independence ensures that the measurements of each factor are not influenced by the measurements of the other factors and their effects on the overall indicator can be isolated, measured separately and accurately quantified. Another criterion is to control for *actionability* as defined by Meneely et al. (2010), meaning that the indicator provides recommendations for action and enables the institute to make informed decisions based on the indicator's outcome (Roche, 1994). We will examine for d*efinition validity* meaning that the indicator's definition is clear and unambiguous, and its measurements are consistent across all who implement it (Lincke and Löwe, 2006). The indicator will also be checked for the possibility to exhibit *unexpected discontinuities* (Kitchenham et al., 1995) in case the researchers have been idle in relation to open science, or if they have not published any article (denominators are equal to zero). *Transferability* (Roche, 1994) is another criterion that is examining the potential of the indicator to be transferred to domains other than Pure.

Eisinga et al. (2013) claim that to measure the *reliability* of a two-factor indicator, the Spearman-Brown formula is deemed more appropriate (in relation to Cronbach's alpha, for example, that underestimates the reliability to a great extent). The Spearman-Brown correlation is a nonparametric measure that neither assumes that the data are normally distributed nor that their relationship is linear. Therefore, it is ideal for our dataset. The normality of the indicator's distributions across the departments will then be examined following the correlation values mentioned by Ahdika (2017).

For all calculations we will use the R free software environment for statistical computing and graphics as well as a number of R supported packages (Warrens, 2016; Wickham, 2016; Pronk et al., 2021; R Core Team, 2022; Kassambara, 2023).

## Results

**Figure 2:**
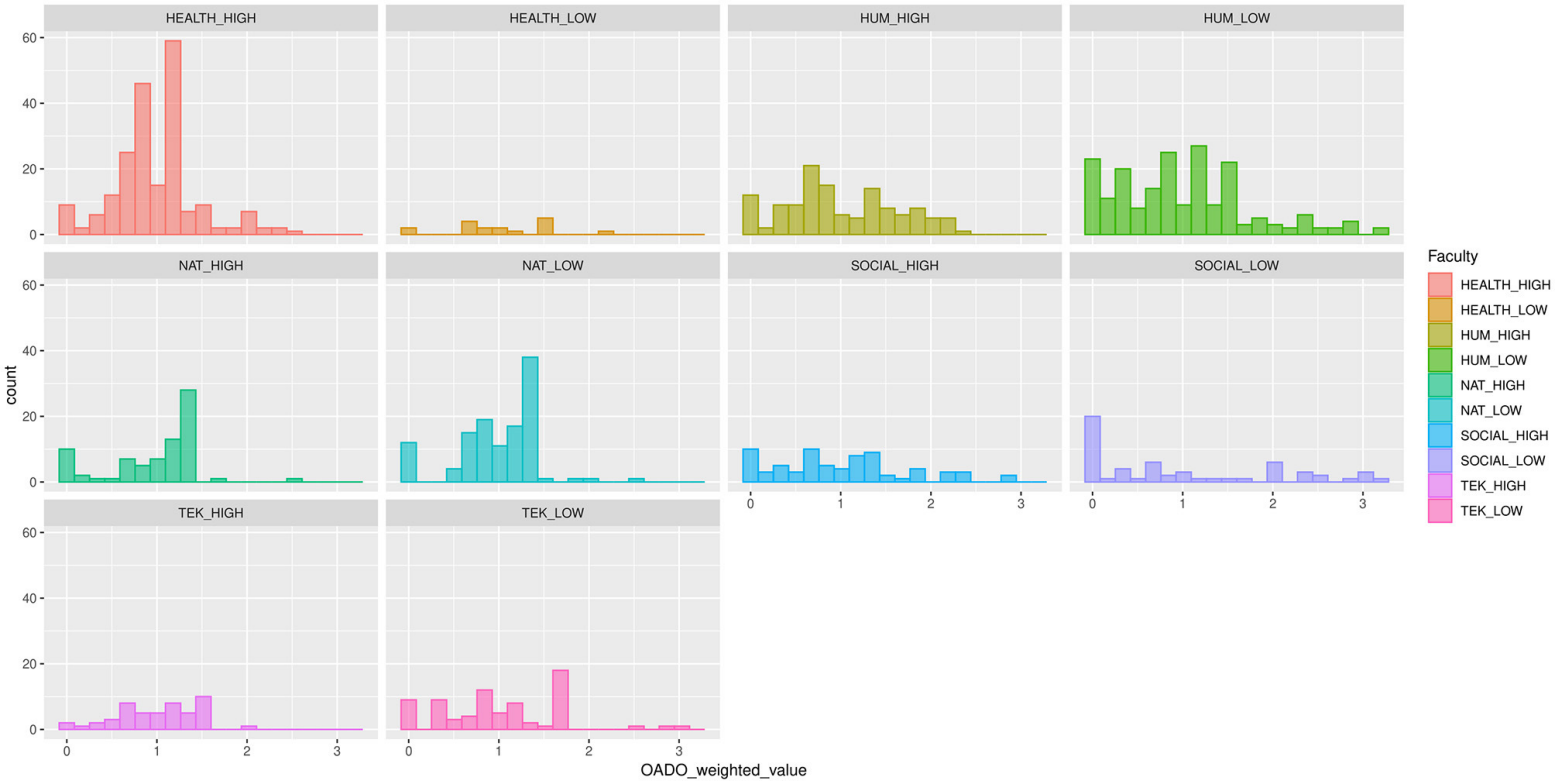
Histograms of the Weighted-OADO values of 995 researchers grouped by faculty and department. Each faculty is represented by two departments, the one that performs the highest in open access and the one that performs the lowest.

Figure 2 reveals the shape and spread of the histograms that display the distribution of the Weighted-OADO values for all the 995 researchers grouped by faculty. Each faculty is represented by two departments, the best one (HIGH) in relation to open access and the worst one (LOW) as they appear at the Danish Open Access Indicator. All researchers with Weighted-OADO value below 1 are less open in relation to their department average, and all researchers with Weighted-OADO value over 1 are more open in relation to their department average. Table 1 summarizes the characteristics of the dataset providing mean, median, min, max and standard deviation values.

**Table 1:** Descriptive statistics.

**Faculty**	**Mean**	**Max**	**Min**	**Median**	**Std**
HEALTH_HIGH	1	2.5	0	1	0.471
HEALTH_LOW	1	2.2	0	1	0.572
HUM_HIGH	1	2.3	0	0.9	0.632
HUM_LOW	1	3.2	0	0.9	0.744
NAT_HIGH	1	2.5	0	1.1	0.518
NAT_LOW	1	2.5	0	1	0.465
SOCIAL_HIGH	1	2.9	0	0.95	0.738
SOCIAL_LOW	1	3.2	0	0.7	1.06
TEK_HIGH	1	2.1	0	1	0.447
TEK_LOW	1	3	0	0.95	0.673

**Figure 3:**
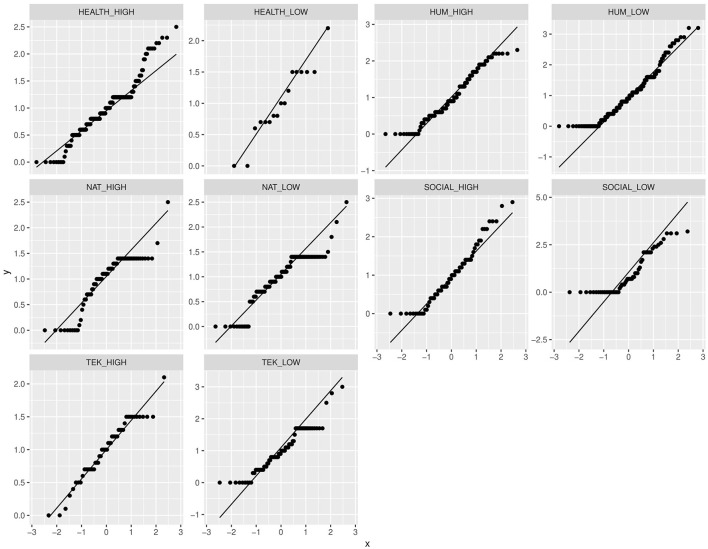
The quantile-quantile plots of the faculties and departments.

The Q-Q plots of the departments are illustrated in Figure 3. We observe that all Weighted-OADO distributions appear to be skewed apart from the HEALTH_LOW and TEK_HIGH where most of the quantile points lie along the straight diagonal line. As the Shapiro-Wilk test for normality indicates, indeed only these two departments are normally distributed as they have *p* > 0.05 (HEALTH_LOW: *p* = 0.315, and TEK_HIGH: *p* = 0.0958).

Table 2 shows the Spearman-Brown correlation of the research and communication OADO factors per faculty and department, and their levels of reliability. Only the departments of HEALTH_LOW and TEK_HIGH seem to be moderately internally consistent, meaning that there is some variability in the values that is not due to the construct being measured. Regarding the direction of the relationship of the two factors, we observe that the departments HEALTH_HIGH, NAT_HIGH, NAT_LOW, TEK_HIGH and TEK_LOW have a negative correlation which implies that research and communication factors vary in opposite directions, that is, when the research factor increases, the communication factor decreases and vice versa. Regarding the strength of the relationship: The more extreme the correlation coefficient (closer to −1 or 1), the stronger the relationship. This also means that a correlation close to zero indicates that the two variables are independent, that is, as one variable increases, there is no tendency in the other variable to either decrease or increase.

**Table 2:** Spearman-Brown levels of reliability for the two OADO factors per faculty and department.

**Faculty**	**Spearman-Brown correlation between the research and communication OADO factors**	**Reliability (Ahdika, 2017)**
HEALTH_HIGH	−0.223	Rather reliable
HEALTH_LOW^*^	0.419	Quite reliable
HUM_HIGH	0.175	Less Reliable
HUM_LOW	0.106	Less Reliable
NAT_HIGH	−0.0862	Less Reliable
NAT_LOW	−0.0369	Less Reliable
SOCIAL_HIGH	0.332	Rather reliable
SOCIAL_LOW	0.283	Rather reliable
TEK_HIGH^*^	−0.492	Quite reliable
TEK_LOW	−0.176	Less Reliable

^*^Most reliable departments.

The implementation set-up as described in the methods section is not complicated; thus, it should be possible to apply it to other institutes using Pure. Institutes that are not using Pure could also use the OADO with slight modifications, as long as OA is managed, or registered in the local RIMS. In general, it is possible to use the OADO in platforms where the OA status is findable and where there is coverage of both research and outreach publications. Thus, the indicator has transferability. In addition, the indicator could also use data from curated abstract and citation databases like Scopus, although this would shift the focus of the OADO to research publications over communication publications.

It is possible for the OADO to exhibit unexpected discontinuities if the researchers have been academically idle during the selected period, that is if they have not published any article (denominators equal to zero), or if they have not practiced open science (numerators equal to zero) even though they may have been academically active. In contrast to the h-index that always increases even when the researcher is inactive, the OADO will fall to zero in such case.

The OADO has actionability as it provides recommendations for action and enables the institute to make informed decisions based on the indicator's outcome, for example recognize and reward open science, and change the systems of evaluation. The OADO, additionally, has definition validity meaning that its measuring what is intended to measure based on the theoretical concept it is representing.

## Discussion

In academia, as in the rest of society today, most things are counted and measured. What is counted can be rewarded, and concurrently what cannot be counted is usually not rewarded. The OADO indicator gives an opportunity to consider OS as a non-citation-based metric not dependent on the degree of indexing in a citation database. Altmetric (Adie and Roe, 2013) and PlumX (Champieux, 2015) -referring to the “Mentions” and “Social Media” categories-are collections of attention of specific research outputs, though they are not based on academic citations but rather mentions in various SoMe, news outlets and patent-registers. These are commendable as alternatives to traditional citation-based metrics, but they are less efficient at harvesting non-English language outlets. They are also not an index, but a range of counts and typically based not on the researchers, but rather on publication outputs. Therefore, their outreach perspective cannot be compared to that of the OADO.

The OADO indicator proposed is a new indicator which is citation-database independent and can be used at an individual researcher level or at a more aggregate level to elucidate the openness of scholarly output relative to their comparable group of peers. The indicator is computed using the local RIMS. This means it can be implemented at any university or research institution using a RIMS which registers OA status. This also means the researchers on whom the indicator is calculated can take on an active role and ownership in the calculation of the indicator.

The results indicate that the OADO is a reliable indicator for normally distributed departments, in our case HEALTH_LOW and TEK_HIGH. From Figure 1, we notice that these two departments have the lowest number of researchers, 17 and 50, respectively. We may infer that each of them perhaps consists of very few research groups or centers which are probably committed to specific branches of research in their discipline, following similar discipline-specific methods, traditions and codes of conduct. Thus, the researchers of small departments seem to approach OS in the same manner. Further research is needed, but from our results so far, we could suggest that in larger departments the OADO should be applied per research group or center.

The negative correlation between the two OADO factors, especially for the departments of TEK_HIGH and HEALTH_HIGH that had the highest value, perhaps requires further qualitative investigation. It may be an indication that the departments have a policy in place to write open communication publications when OA is blocked, or an indication of a deluded belief that further dissemination of a study is not necessary as long as the original article was published OA.

For institutions or countries with policies in place that aim to increase the level of openness, management needs valid information to support strategy discussions. The management of research units that have an interest in researchers' efforts to make their publications and data openly available will be provided with an indicator assisting them in decision making and strategy writing. Since the Weighted-OADO can be used within a department, it is possible for the department to reward researchers that to a higher degree make their publications and data open compared to their near colleagues. Those near colleagues are likely to be in the same or a related research field, thus making the comparison fairer. Furthermore, it is also possible to take into account specificities within the department, if say, a department wants to value open outreach further, the a and b weights of the OADO function can be adjusted locally to help emphasize this.

Reflecting on the study, few noteworthy limitations that should be mentioned follow.

- A researcher's Weighted-OADO is calculated based on publications that can be affected by incomplete registration in RIMS. It is therefore important to support data registration to the local RIMS as much as possible.
- Not all researchers will add publications from their entire career to the local RIMS. In fact, some universities reach the extent to forbid researchers to add publications with previous affiliations. Further, with the RIMS systems precision getting constantly improved, limiting oneself to a smaller span of years, gives a more precise and current indication of OS allowing a researcher to be rewarded (or not) for a changed approach in more recent years.
- The option of adding datasets to the RIMS system is rather new and neither yet widely used, nor widely advocated, thus constituting another limitation. As the degree of open datasets stands at SDU, the OD part of the OADO might right now have a lot < 100% coverage. This might not be the case, of course, in other universities. The OADO is still usable, and we expect OD to soon be implemented to a higher degree, as the demand for datasets following the FAIR principles is rising steeply.
- Elaborating on the fact that what can be considered as a “good” Weighted-OADO score would vary from 1 up to 3.2 in our case (see Table 1) and that this score is bound to the OADO scores of the researchers comprising each department, unhealthy comparisons may be initiated between researchers from different departments. There may be the case, for example, of a researcher practicing OS very actively from a department that is also very active in OS having a lower Weighted-OADO score than a researcher who is less active in OS in a department with less OS practices. Thus, comparing the Weighted-OADO of these two researchers would not make any sense.
- Lastly, the OADO can be calculated based on any number of publications and does not favor those who have published a lot unlike other indicators such as the h- and g-indices, along with various counts of outputs and citations. Researchers with a lower number of publications will have a greater impact on their OADO score by making one publication OA, one dataset open and one communication article open in relation to researchers with a higher number of publications. However, limiting the time span the OADO calculation is based on will keep the potential publications number low and easier to impact for all researchers, compared to indicators that include their output in their entirety.

## Conclusion

We have presented a new person-centered indicator (the OADO) that we propose may be used to evaluate the openness of an individual researcher regarding his/her research and communication output relative to their comparable disciplinary group of peers. The OADO shows only the degree of OS, as there are other indicators to show the span of publication numbers and citations during a researcher's career. The indicator is computed based on data available in a typical Research Information Management System, allowing it to be implemented at a university, or a similar research institution. We have tested the OADO against a set of validation criteria with data from our own university and conclude that it is a reliable indicator for normally distributed departments. Hence, before employing this indicator it is important to check the distribution of the Weighted-OADO. However, in cases when the normality assumption is not satisfied, the indicator may be useful as to indicate the need for actions to increase openness, e.g., pointing toward the need for OS policy measures, and incentives or systematic registration of research outputs.

## Data availability statement

The datasets presented in this study can be found in online repositories. The names of the repository/repositories and accession number(s) can be found at: Zenodo, https://zenodo.org/record/8082868.

## Author contributions

EV contributed to conception, design of the study, analysis, visualization, reflection, data availability, and drafted the initial manuscript. TD contributed to conception, state-of-the-art background, and reflection. RE contributed to conception, and data collection. BD contributed to conception and supervision. All authors contributed to manuscript revision, read, and approved the submitted version.

## Footnotes

^1^Council of the European Union (2023), Draft Council conclusions on high-quality, transparent, open, trustworthy and equitable scholarly publishing- Presidency text, https://data.consilium.europa.eu/doc/document/ST-5997-2023-INIT/en/pdf.

^2^The COARA Agreement on Reforming Research Assessment, https://coara.eu/agreement/the-agreement-full-text/.

^3^Pure, https://www.elsevier.com/solutions/pure.

^4^The Danish Open Access Indicator, https://ufm.dk/en/research-and-innovation/cooperation-between-research-and-innovation/open-access/Publications/open-access-barometer.

^5^SDU Open Science Policy, https://sdunet.dk/en/research/library-services/research-data-management-support/the-sdu-open-science-policy.

^6^Unpaywall, https://support.unpaywall.org/support/solutions/articles/44002063718-what-is-an-oa-license- [Accessed March 29, 2023].
